# *Kaempferia galanga* L.: Progresses in Phytochemistry, Pharmacology, Toxicology and Ethnomedicinal Uses

**DOI:** 10.3389/fphar.2021.675350

**Published:** 2021-10-19

**Authors:** Si-Yu Wang, Hui Zhao, Hong-Tao Xu, Xiao-Dong Han, Yun-Shan Wu, Fang-Fang Xu, Xiao-Bo Yang, Ulf Göransson, Bo Liu

**Affiliations:** ^1^ The Second Clinical Medical College, and Guangdong Provincial Key Laboratory of Clinical Research on Traditional Chinese Medicine Syndrome, Guangzhou University of Chinese Medicine, Guangzhou, China; ^2^ Guangzhou Key Laboratory of Chirality Research on Active Components of Traditional Chinese Medicine, Guangzhou, China; ^3^ College of Basic Medical Science, Guangzhou University of Chinese Medicine, Guangzhou, China; ^4^ Shanghai Institute for Advanced Immunochemical Studies, ShanghaiTech University, Shanghai, China; ^5^ State Key Laboratory of Dampness Syndrome of Chinese Medicine, Guangzhou, China; ^6^ Guangdong-Hong Kong-Macau Joint Lab on Chinese Medicine and Immune Disease Research, Guangzhou University of Chinese Medicine, Guangzhou, China; ^7^ Division of Pharmacognosy, Biomedical Center, Uppsala University, Uppsala, Sweden

**Keywords:** *Kaempferia galanga* L., ethnomedicinal uses, phytochemistry, pharmacology, toxicology

## Abstract

*K. galanga* is an aromatic medicinal herb. It is locally to India and distributed in China, Myanmar, Indonesia, Malaysia, and Thailand. *K. galanga* is a Traditional Chinese Herb Medicine (TCHM), which has been applied to treat cold, dry cough, toothaches, rheumatism, hypertension and so on. In addition, it has been used widely as spices since its highly aromas. The aim of this review is to compile and update the current progresses of ethnomedicinal uses, phytochemistry, pharmacology and toxicology of *K. galanga*. All the data on *K. galanga* were based on different classical literary works, multiple electronic databases including SciFinder, Web of Science, PubMed, etc. The results showed that ninety-seven compounds have been identified from rhizome of *K. galanga*, including terpenoids, phenolics, cyclic dipeptides, flavonoids, diarylheptanoids, fatty acids and esters. Modern pharmacology studies revealed that extracts or secondary metabolites of the herb possessed anti-inflammatory, anti-oxidant, anti-tumorous, anti-bacterial, and anti-angiogenesis effects, which were closely related to its abundant ethnomedicinal uses. In conclusion, although previous research works have provided various information of *K. galanga*, more in-depth studies are still necessary to systemically evaluate phytochemistry, pharmacological activities, toxicity and quality control of this herb.

## Introduction

*K. galanga* is from a dried rhizome of herb *Kaempferia galanga* L., belonging to the important family Zingiberaceae and genus *Kaempferia*. It also called sand ginger, aromatic ginger in different areas. *K. galanga* is native to India, and commonly found in China, Myanmar, Indonesia, Malaysia, and Thailand (Wang and Tang, 1980; Santhoshkumari and Devi, 1991). In Southern China, such as Guangxi, Guangdong, Yunnan are the main producing areas of *K. galanga*. In China, being a source of valuable bioactive compounds, it was used as a folk medicine due to its good curative effect on rheumatism, dry cough, colic, muscle pain, inflammations, as well as tumors (Park et al., 2005; Liu et al., 2010; He et al., 2012). In India, *K. galanga* was used in the treatment of intestinal wounds and urticarial (Nazar et al., 2008; Seth and Maurya, 2014). In Malaysia, *K. galanga* was also applied for abdominal pain and postpartum care in woman (Hirschhorn, 1983). Moreover, it could also be used as food condiment. According to Sivarajan and Balachandran (1994), *K. galanga* was used to treat phlegm, fever, cough, meanwhile, it also exerts good effect as a diuretic, anabolic, and carminative.

**FIGURE 1:**
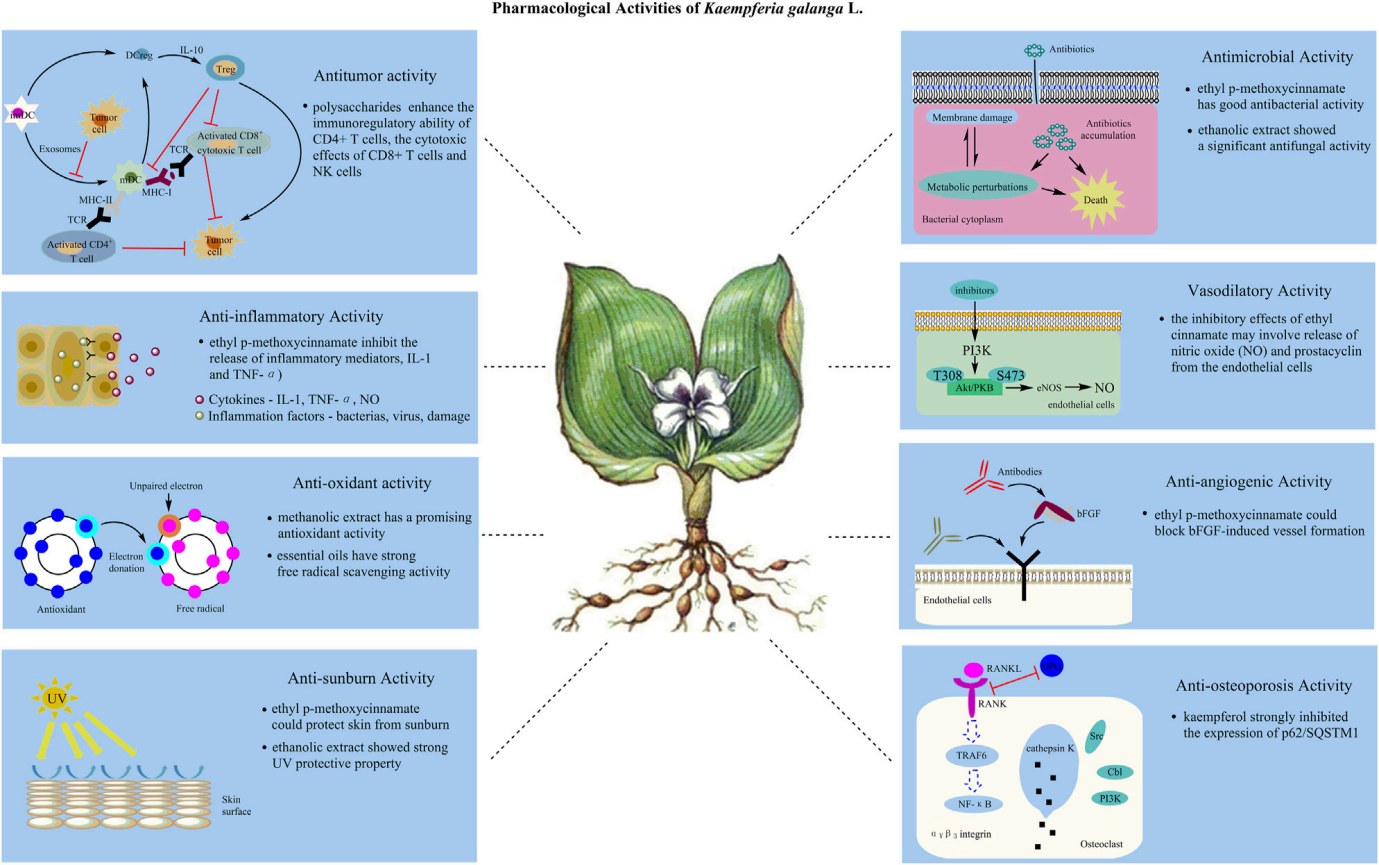
Primary reactions and constituents supporting various biological effects of *K. galanga*. The TCHM shows antitumor, anti-inflammatory, anti-oxidant, anti-sunburn, antimicrobial, vasodilatory, anti-angiogenic and anti-osteoporosis activities, et al. Multiple types of constituents (see Table 3) are contributed to such evident pharmacological profiles of this plant.

To date, phytochemical studies have discovered many chemical compounds of the plant, mainly terpenoids, phenolics, diarylheptanoids and flavonoids. Also, it has revealed that the components or extracts from *K. galanga* exhibit anti-inflammatory, anti-oxidant, anti-tumorous, anti-angiogenesis, and other effects in Figure 1 (Umar et al., 2014; Wu et al., 2015; Zhou et al., 2015; Yao et al., 2018; Srivastava et al., 2019). However, pharmacological researches mainly focus on the crude extracts and characteristic compounds especially *trans* ethyl p-methoxycinnamate. Furthermore, many active components in the extracts of *K. galanga* have not been fully investigated yet as well as the mechanisms of action. In addition, biological evaluations should take appropriate effective dose, the frequency of administration and duration of treatment into consideration. Thus, there are many issues worthy of further study.

Although previous reports provided great inspiration and help for us (Umar et al., 2011; Munda et al., 2018; Elshamy et al., 2019; Kumar, 2020), we are more concerned about the application of *K. galanga* in ethnomedicine, its relationship with phytochemistry, modern pharmacology, and its toxicity. Herein, we conducted a comprehensive review on the phytochemistry, pharmacology, toxicology and ethnomedicinal uses of *K. galanga.* We also discuss the limitations of the current studies of the herb and suggest areas of interest for potential future research. We hope to provide valuable information for future in-depth investigations and applications of the herb.

## Review Methodology

The literature for this review was collected from different classical literary works, multiple electronic databases including SciFinder, Web of Science, PubMed, Science Direct, Wiley, Springer, CNKI, and PhD, MSc dissertations in Chinese and Pharmacopoeias prior to December 2020 on phytochemistry, pharmacology, toxicology and ethnomedicinal uses of *K. galanga*. A total of 97 publications were collected after preliminary screening, among them, 24 publications used for traditional uses, 34 publications used for phytochemistry, 39 publications used for pharmacological activities. The search terms “*Kaempferia galanga* L” and “*K. galanga* essential oils” were used with no exact time limit. Identify potential full-texts of eligible papers, and check additional and unpublished citations for all relevant references.

## Ethnomedicinal Uses

*K. galanga* has been considered as an important herbal medicine with a long history in China, on the basis of its wide spectrum of biological activities. *K. galanga* was listed in the Chinese medical classic “Compendium of Materia Medica” (Ming dynasty), and it had a good effect on the treatment of pains and cold-damp dysentery. According to the Pharmacopoeia of the People’s Republic of China, *K. galanga* is pungent, warm natured in flavor and belonging to the stomach meridian, and has the action of activating Qi, warm interior, remove digestion and relieve pain.

In addition, *K. galanga* showed significant increase in urine volume and also increased level of sodium and potassium in urine which proves as a strong diuretic agent. Therefore, the results provided a quantitative basis to explain the traditional folkloric use of *K. galanga* as a diuretic agent (Mohammad et al., 2016).

The traditional methods of *K. galanga* are to decoct in water or mash for external use, and a dose of 6–9 g for oral medication is recommended by the Chinese Pharmacopoeia (China Pharmacopoeia Commission, 2015). In addition, although *K. galanga* was widely used, there were few studies on its side effects. The ethnomedicinal uses of *K. galanga* are listed in Table 1.

**TABLE 1:** Ethnomedicinal uses of *K. galanga.*

Locality	Traditional uses	Part used	Method of preparation	References
India	Pain (chest pain, cholera, headache, toothache and abdominal pain)	Rhizome	Rhizomes used as much	Vittalrao et al. (2011); Tewtrakul et al. (2005)
India	Diarrhoea	Rhizome	Intact part used for the management of diarrhoea	Dash et al. (2014)
India	Ulcer	Rhizome	Intact parts used as much	Ogiso and Kobayashi (1994)
Manoko in West Java	Inflammation	Rhizome	Consuming herb tea of this plant rhizome	Levita et al. (2015)
Thailand	Ophthalmia	Leaf	Leaves are used for ophthalmia, Fever and sore throat in the form of lotions and poultices	Kanjanapothi et al. (2004); Warrier et al. (1995)
Malaysia	Swelling and muscular rheumatism	Rhizome	Rhizomes of this plant are boiled with other roots to treat Swelling and muscular rheumatism	Mustafa et al. (1996)
Malaysia	Sore throat	Leaf	The air dried powdered leaves (40 g) soaked in distilled water (1:10; w/v)	Sulaiman et al. (2008)
Thailand	Indigestion, colds	Rhizome	The extract of rhizome	Kanjanapothi et al. (2004)
Japan	Smooth muscle relaxant	Rhizome	Rhizomes used as smooth muscle relaxant	Hashimoto et al. (1986)
Malaysia	Swollen breasts, coughs	Leaf	The ashes of leaves are rubbed on swollen breasts after childbirth while fresh leaves are chewed for relieving coughs	Sulaiman et al. (2008)
Indonesia	Osteoarthritis	Rhizome	Intact part used for treatment for osteoarthritis	Akmal et al. (2017)
Indonesia	Recurrent aphthous stomatitis (RAS)	Rhizome	Rhizome’s extract is effective in treating minor RAS	Laurenzia and Wilda (2016)
Thailand	Cardiotonic	Rhizome	Rhizomes used as cardiotonic and central nervous system (CNS) stimulants	Mokkhasmit et al. (1971)
Malaysia	Hypertension	Rhizome	Intact part used for treating Hypertension	Othman et al. (2002)
India	Hepatoprotection	Rhizome	Rhizome’s constituents have promising application in hepatoprotection	Manigaunha et al. (2010)
India	Hypolipidemia	Rhizome	Rhizomes extracts shows significant activity for treating hypolipidemic	Achuthan and Padikkala (1997)
Malaysia	Tumor	Rhizome	Rhizomes used as much	Omar et al. (2017)
India	Washing hairs	Leaf	Leaves are used as a perfume in washing hairs	Warrier et al. (1995)
Bangladesh	Pregnancy	Leaf	Leaf infusions can be used as a beneficial drink for women	Rahman et al. (2004)
China	Dyspepsia	Rhizome	Rhizomes have been used as an aromatic stomachic to promote digestion	China Pharmacopeia Commission (2015)
China	Anxiety	Rhizome	Its aroma has also been used for a long history in relieving anxiety	He et al. (2012)

## Phytomedicinal Formulations

*K. galanga* has been used as a phyto-ingredient in some classical medicinal formulations. It was combined with other herb to treat common pains, cold, digestive disorders as formulations, and these formulations could be made into different dosage forms or decocted with water depending on the maximum efficacy to use them. (Kanjanapothi et al., 2004). The traditional formulations containing *K. galanga* are listed in Table 2.

**TABLE 2:** Classic prescriptions of *K. galanga.*

Formulations	Uses	Mode of uses	Locality	References
*Quercus infectoria*, *Glycyrrhiza uralensis*, *Kaempferia galanga* and *Coptis chinensis*	Four plant powders are consisting of traditional Thai herbal remedy for aphthous ulcer	Powders (oral)	Thailand	Aroonrerk and Kamkaen (2009)
*Kaempferia galanga* L (Kencur) and *Boesenbergia pandurata* (Roxb) Schlecht (Temu kunci)	Combination of kencur and temu lawak ethanol extract with ratio (80%:20%) or (70%:30%) as Sunscreen	Cream (external use)	Southeast Asia, such as Indonesia and Thailand	Shintia et al. (2018)
*Plumbago indica*, *Garcinia mangostana*, *Dracaena loureiri*, *Dioscorea membranacea*, *Artemisia annua*, *Piper chaba*, *Myristica fragrans* and *Kaempferia galanga*	Eight powdered medicinal plants showed potent antimalarial activity	Powders (oral)	Thailand	Thiengsusuk et al. (2013)

## Toxicology

Although *K. galanga* has long been used as TCHM, its systematic toxicity and safety evaluations are still unclear. The acute and subacute toxicity tests of its rhizomes ethanol extract (maximum single oral dose up to 5,000 mg/kg (b. w.), and daily dose of 1,000 mg/kg (b. w.) for 30 consecutive days) showed that it has no significant toxicity regarding to the morbidity and mortality (Amuamuta et al., 2017).

Similarly, Kanjanapothi et al. have reported that the maximum tolerated dose (MTD) of ethanol extract of rhizomes of *K. galanga* was up to 5,000 mg/kg and no death occurred in rats by oral administration. Hematological analysis showed no difference in any parameter tested between control and test group in male and female. Moreover, no abnormal in pathology and histopathology, and no irritation in the skin. Besides, in 28 days subacute toxicity studies, there was no death occurred when the ethanolic *K. galanga* extract was treated the dosage of 25, 50 or 100 mg/kg (Kanjanapothi et al., 2004). Therefore, *K. galanga* is safe for the vital organs during treatment depending on the above toxicological studies.

## Phytochemistry

**FIGURE 2:**
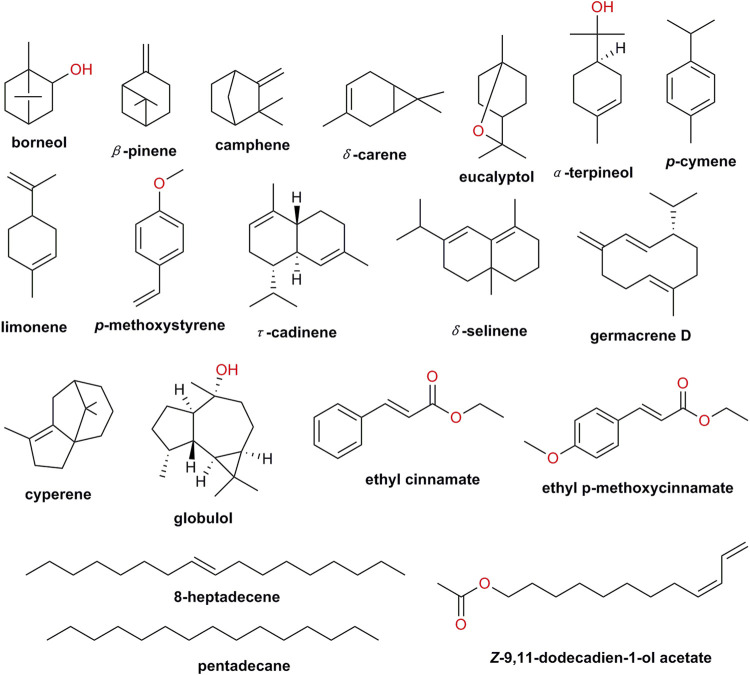
Chemical structures of main volatile components in *K. galanga*.

**FIGURE 3:**
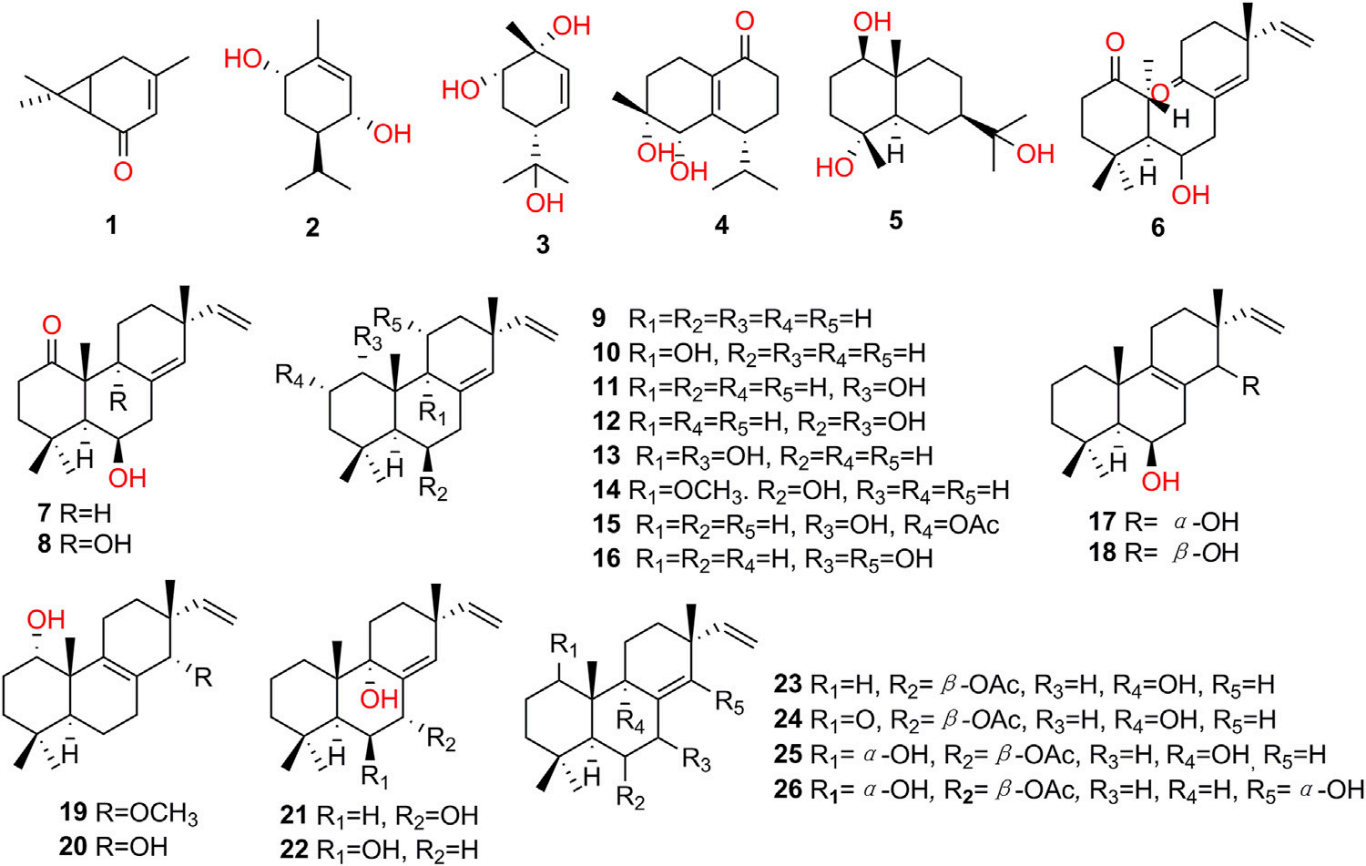
Structures of terpenoids isolated from *K. galanga*.

**FIGURE 4:**
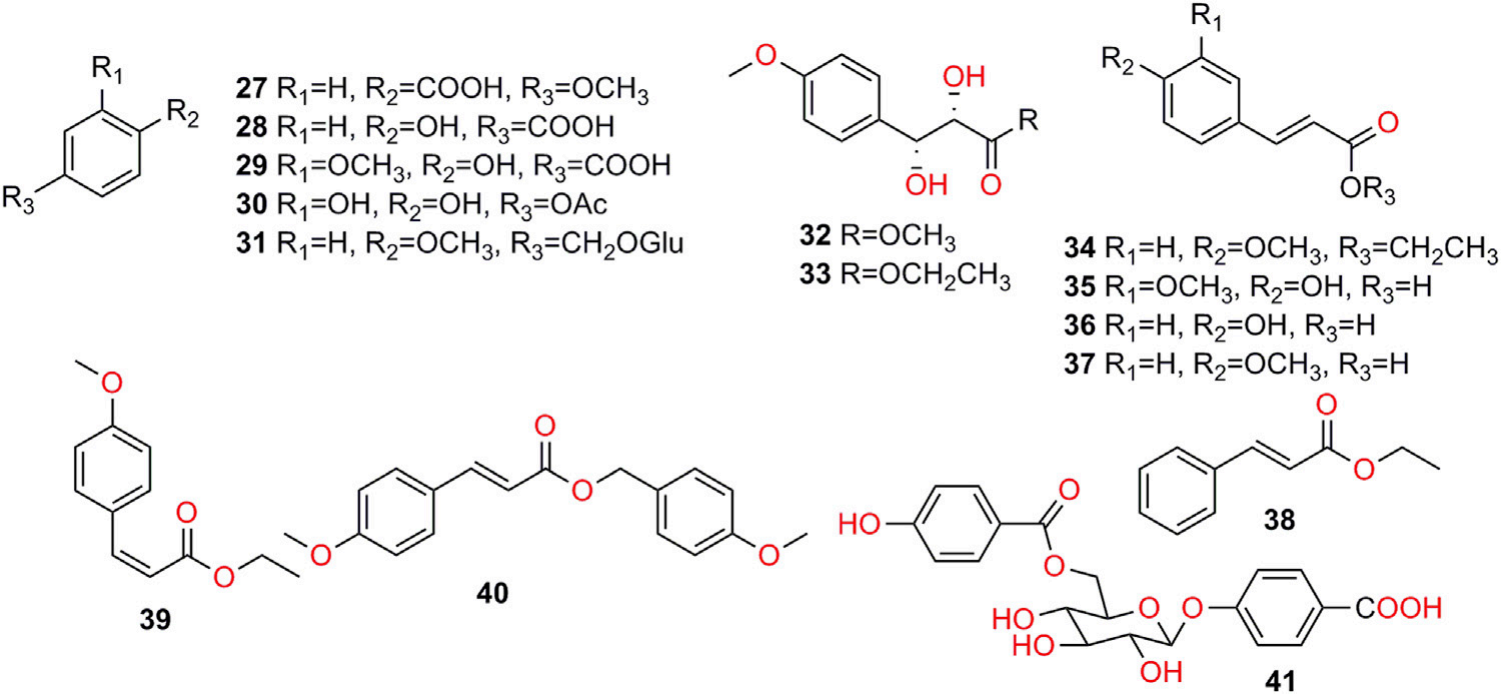
Structures of phenolics isolated from *K. galanga*.

**FIGURE 5:**
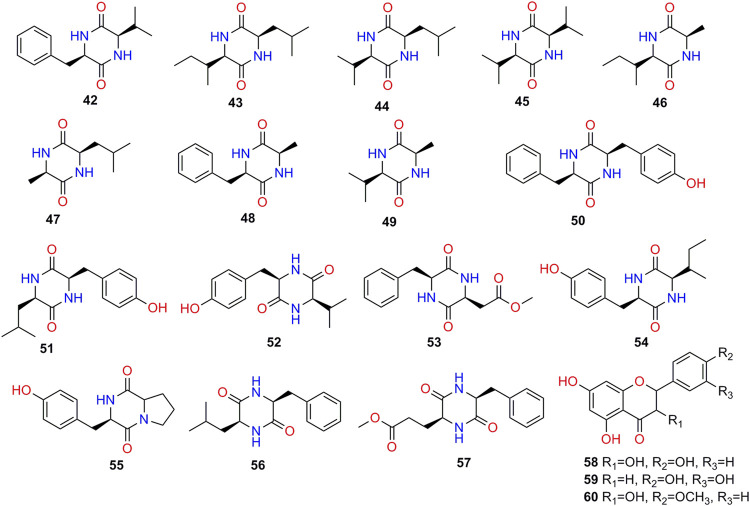
Structures of cyclic dipeptides and flavonoids isolated from *K. galanga*.

**FIGURE 6:**
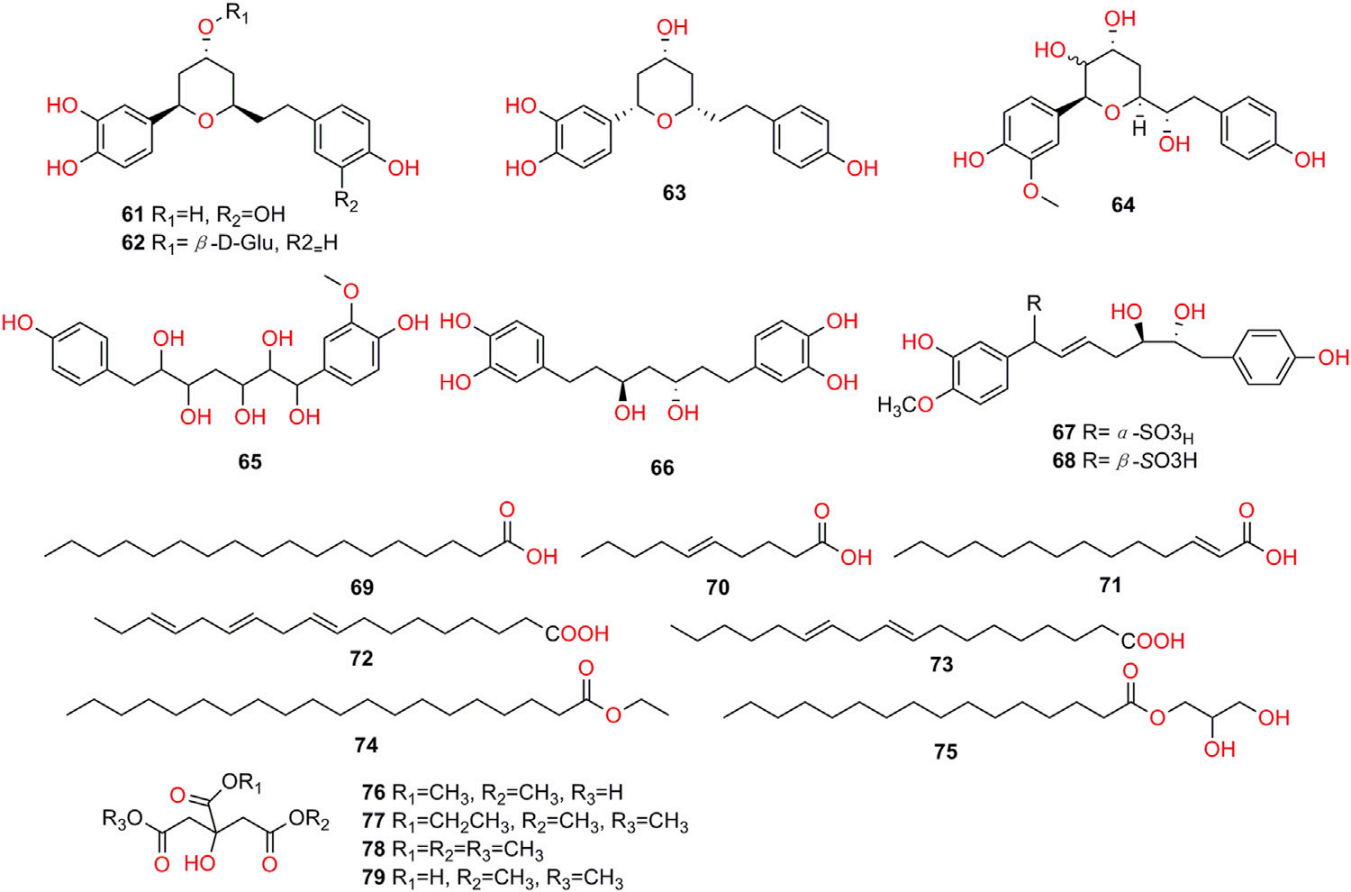
Structures of diarylheptanoids, fatty acids and esters isolated from *K. galanga*.

**FIGURE 7:**
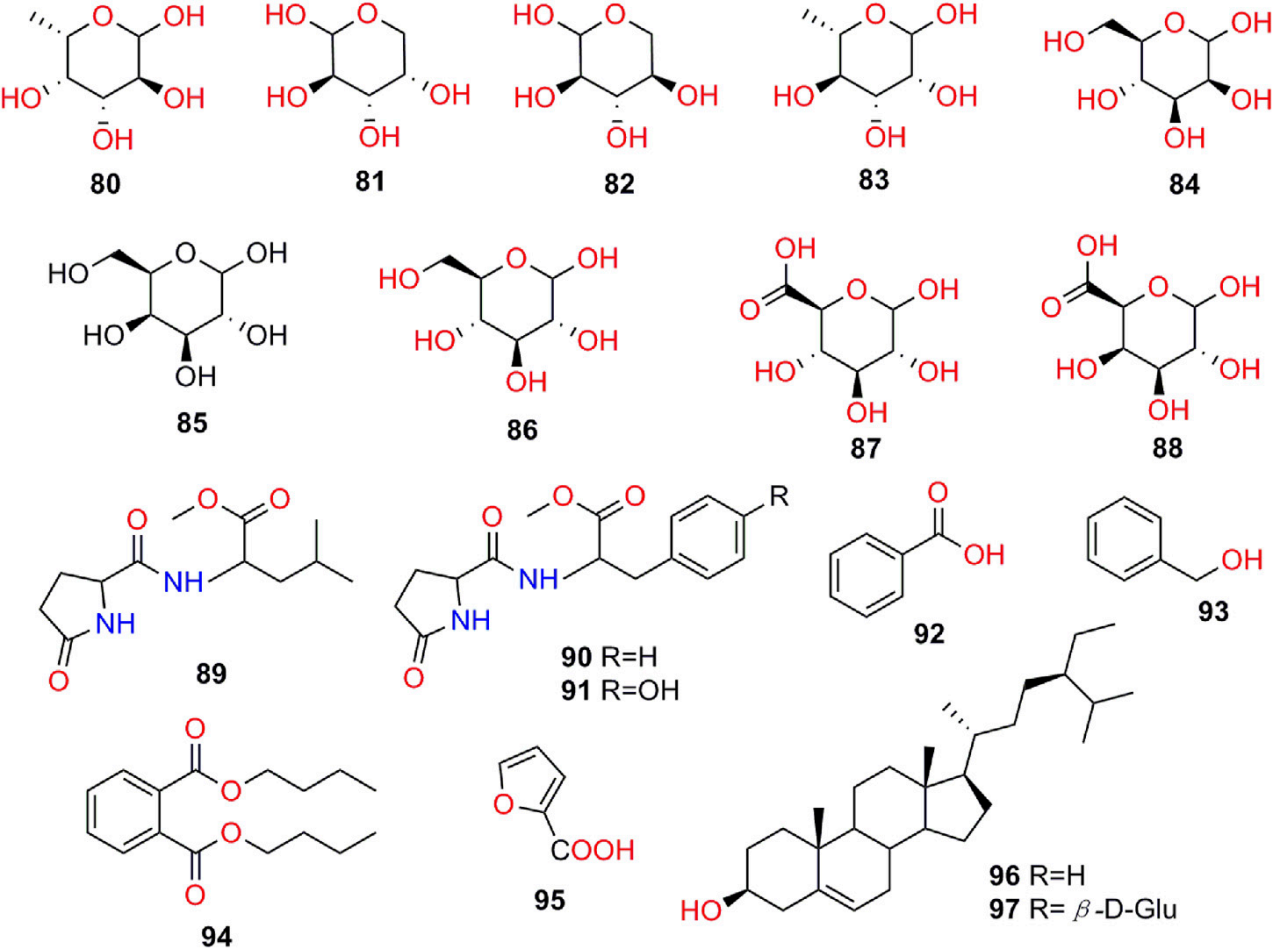
Structures of polysaccharides and other compounds isolated from *K. galanga*.

Chemical characteristics of *K. galanga* showed the existence of various types of secondary metabolites such as terpenoids, phenolics, cyclic dipeptides, diarylhaptanoids, flavonoids, polysaccharides, and essential oils. A total of 97 compounds have been obtained from the rhizome of *K. galanga*. In this article, we will present each types of compounds in Table 3, and structures in Figures 2-7.

**TABLE 3:** Chemical constituents isolated from *K. galanga*.

No	Chemical component	Chemical formula	References
**Terpenoids**			
**1**	3-caren-5-one	C_10_H_14_O	Kiuchi et al. (1987)
**2**	(3*R*,4*R*,6*S*)-3,6-dihydroxy-1-menthene	C_10_H_18_O_2_	Yao (2018)
**3**	(1*R*,2*S*,4*R*)-p-menth-5-ene-1,2,8-triol	C_10_H_18_O_3_	Yao (2018)
**4**	oxyphyllenodiol B	C_14_H_22_O_3_	Yao (2018)
**5**	hedytriol	C_15_H_28_O_3_	Yao (2018)
**6**	kaemgalangol A	C_20_H_30_O_3_	Ningombam et al. (2018)
**7**	6β-hydroxypimara-8(14),15-diene-1-one	C_20_H_30_O_2_	Ningombam et al. (2018)
**8**	sandaracopimaradien-6β,9α-diol-l-one	C_20_H_30_O_3_	Ningombam et al. (2018)
**9**	(-)-sandaracopimaradiene	C_20_H_32_	Ningombam et al. (2018)
**10**	sandaracopimaradiene-9α-ol	C_20_H_32_O	Ningombam et al. (2018)
**11**	kaempulchraol I	C_20_H_32_O	Ningombam et al. (2018)
**12**	kaempulchraol E	C_20_H_32_O_2_	Ningombam et al. (2018)
**13**	8(14),15-sandaracopimaradiene-1α,9α-diol	C_20_H_32_O_2_	Ningombam et al. (2018)
**14**	kaempulchraol L	C_21_H_34_O_2_	Ningombam et al. (2018)
**15**	2α-acetoxy sandaracopimaradien-1α-ol	C_22_H_34_O_3_	Ningombam et al. (2018)
**16**	1,11-dihydroxypimara-8(14),15-diene	C_20_H_32_O_2_	Ningombam et al. (2018)
**17**	6β,14α-dihydroxyisopimara-8(9),15-diene	C_20_H_32_O_2_	Tungcharoen et al. (2020)
**18**	6β,14β-dihydroxyisopimara-8(9),15-diene	C_20_H_32_O_2_	Tungcharoen et al. (2020)
**19**	1α-hydroxy-14α-methoxyisopimara-8(9),15-diene	C_21_H_34_O_2_	Tungcharoen et al. (2020)
**20**	1α,14α-dihydroxyisopimara-8(9),15-diene	C_20_H_32_O_2_	Tungcharoen et al. (2020)
**21**	boesenberol I	C_20_H_32_O_2_	Ningombam et al. (2018)
**22**	boesenberol J	C_20_H_32_O_2_	Ningombam et al. (2018)
**23**	6β-acetoxysandaracopimaradiene-9α-ol	C_22_H_34_O_3_	Tungcharoen et al. (2020)
**24**	6β-acetoxysandaracopimaradiene-9α-ol-1-one	C_22_H_32_O_4_	Tungcharoen et al. (2020)
**25**	6β-acetoxysandaracopimaradiene-1α,9α-diol	C_22_H_34_O_4_	Tungcharoen et al. (2020)
**26**	6β-acetoxy-1α-14α-dihydroxyisopimara-8(9),15-diene	C_22_H_34_O_4_	Yao (2018)
**Phenolics**			
**27**	p-metho-xybenzoicacid	C_8_H_8_O_3_	Yao et al. (2018)
**28**	p-hydroxybenzoic acid	C_7_H_6_O_3_	Yao et al. (2018)
**29**	vanillic acid	C_8_H_8_O_4_	Yao et al. (2018)
**30**	methyl 3,4-dihydroxybenzoate	C_8_H_8_O_4_	Yao et al. (2018)
**31**	4-methoxybenzyl-O-β-D-glucopyranoside	C_14_H_20_O_7_	Yao et al. (2018)
**32**	methyl (2*R*,3*S*)-2,3-dihydroxy-3-(4-methoxyphenyl) propanoate	C_11_H_14_O_5_	Yao et al. (2018)
**33**	ethyl (2*R*,3*S*)-2,3-dihydroxy-3-(4-methoxyphenyl) propanoate	C_12_H_16_O_5_	Yao et al. (2018)
**34**	*trans* ethyl p-methoxycinnamate	C_12_H_14_O_3_	Yao et al. (2018)
**35**	ferulic acid	C_10_H_10_O_4_	Yao et al. (2018)
**36**	*trans* p-hydroxycinnamic acid	C_9_H_8_O_3_	Yao et al. (2018)
**37**	*trans* p-methoxycinnamic acid	C_10_H_10_O_3_	Yao et al. (2018)
**38**	*trans* ethyl cinnamate	C_11_H_12_O_2_	Wu (2016)
**39**	*cis* ethyl p-methoxycinnamate	C_12_H_14_O_3_	Wu (2016)
**40**	4-methoxy-benzyl (*E*)-3-(4-methoxyp-henyl) acrylate	C_18_H_18_O_4_	Wu (2016)
**41**	1-O-4-carboxylphenyl-(6-O-4-hydroxybenzoyl)-β-D-glucopyranoside	C_20_H_20_O_10_	Yao et al. (2018)
**Cyclic Dipeptides**			
**42**	cyclo-(L-Val-L-Phe)	C_14_H_18_N_2_O_2_	Yao (2018)
**43**	cyclo-(L-Leu-L-Ile)	C_12_H_22_N_2_O_2_	Yao (2018)
**44**	cyclo-(L-Val-L-Leu)	C_11_H_20_N_2_O_2_	Yao (2018)
**45**	cyclo-(L-Val-L-Val)	C_10_H_18_N_2_O_2_	Yao (2018)
**46**	cyclo-(L-Ala-L-Ile)	C_9_H_16_N_2_O_2_	Yao (2018)
**47**	cyclo-(L-Ala-L-Leu)	C_9_H_16_N_2_O_2_	Yao (2018)
**48**	cyclo-(L-Ala-L-Phe)	C_12_H_14_N_2_O_2_	Yao (2018)
**49**	cyclo-(L-Val-L-Ala)	C_8_H_14_N_2_O_2_	Yao (2018)
**50**	cyclo-(L-Phe-L-Tyr)	C_18_H_18_N_2_O_3_	Yao (2018)
**51**	cyclo-(L-Leu-L-Tyr)	C_15_H_20_N_2_O_3_	Yao (2018)
**52**	cyclo-(L-Val-L-Tyr)	C_14_H_18_N_2_O_3_	Yao (2018)
**53**	cyclo-(L-Asp-OCH_3_-L-Phe)	C_14_H_16_N_2_O_4_	Yao (2018)
**54**	cyclo-(L-Tyr-L-Ile)	C_15_H_20_N_2_O_3_	Yao (2018)
**55**	cyclo-(L-Pro-L-Tyr)	C_14_H_16_N_2_O_3_	Yao (2018)
**56**	cyclo-(L-Leu-L-Phe)	C_15_H_20_N_2_O_2_	Yao (2018)
**57**	cyclo-(L-Glu-OCH_3_-L-Phe)	C_15_H_18_N_2_O_4_	Yao (2018)
**Flavonoids**			
**58**	kaempferol	C_15_H_10_O_6_	Wu (2016)
**59**	luteolin	C_15_H_10_O_6_	Wu (2016)
**60**	kaempferide	C_16_H_12_O_6_	Jiao et al. (2017)
**Diarylheptanoids**			
**61**	(1*R*,3*R*,5*R*)-1,5-epoxy-3-hydroxy-1-(3,4-dihydroxyphenyl)-7-(3,4-dihydroxyphenyl) heptane	C_19_H_22_O_6_	Yao et al. (2018)
**62**	(1*R*,3*R*,5*R*)-1,5-epoxy-3-hydroxy-1-(3,4-dihydroxyphenyl)-7-(4-hydroxyphenyl) heptane 3-O-β-D-glucopyranoside	C_25_H_32_O_10_	Yao et al. (2018)
**63**	phaeoheptanoxide	C_19_H_22_O_5_	Yao (2018)
**64**	hedycoropyran B	C_20_H_24_O_7_	Yao (2018)
**65**	1-(4-hydroxy-3-methoxyphenyl)-7-(4-hydroxyphenyl) heptane-1,2,3,5,6-pentaol	C_20_H_26_O_8_	Yao et al. (2018)
**66**	(3*R*,5*S*)-3,5-dihydroxy-1,7-bis(3,4-dihydroxyphenyl) heptane	C_19_H_24_O_6_	Yao (2018)
**67**	kaempsulfonic acid A	C_20_H_24_O_8_S	Wang et al. (2013)
**68**	kaempsulfonic acid B	C_20_H_24_O_8_S	Wang et al. (2013)
**Fatty Acids and Esters**			
**69**	stearic acid	C_18_H_36_O_2_	Wu (2016)
**70**	dec-5-enoic acid	C_10_H_18_O_2_	Wu (2016)
**71**	2-tetradecenoic acid	C_14_H_26_O_2_	Wu (2016)
**72**	linolenic acid	C_18_H_30_O_2_	Yao (2018)
**73**	linoleic acid	C_18_H_32_O_2_	Yao (2018)
**74**	ethyl icosanoate	C_22_H_44_O_2_	Wu (2016)
**75**	monopalmitin	C_19_H_38_O_4_	Wu (2016)
**76**	5,6-dimethyl citrate	C_8_H_12_O_7_	Yao (2018)
**77**	3-carboxyethyl-3-hydroxyglutaric acid 1,5-dimethyl ester	C_10_H_16_O_7_	Yao (2018)
**78**	trimethyl citrate	C_9_H_14_O_7_	Yao (2018)
**79**	1,5-dimethyl citrate	C_8_H_12_O_7_	Yao (2018)
**Polysaccharides**			
**80**	fucose	C_6_H_12_O_5_	Yang et al. (2018)
**81**	arabinose	C_5_H_10_O_5_	Yang et al. (2018)
**82**	xylose	C_5_H_10_O_5_	Yang et al. (2018)
**83**	rhamnose	C_6_H_12_O_5_	Yang et al. (2018)
**84**	mannose	C_6_H_12_O_6_	Yang et al. (2018)
**85**	galactose	C_6_H_12_O_6_	Yang et al. (2018)
**86**	glucose	C_6_H_12_O_6_	Yang et al. (2018)
**87**	glucuronic acid	C_6_H_10_O_7_	Yang et al. (2018)
**88**	galacturonic acid	C_6_H_10_O_7_	Yang et al. (2018)
**Others**			
**89**	L-p Glu-L-Leu-OCH_3_	C_13_H_23_N_2_O_4_	Yao (2018)
**90**	pyroglutamyl-phenylalanine methyl ester	C_16_H_21_N_2_O_4_	Yao (2018)
**91**	pyroglutamyl-tyrosine methyl ester	C_16_H_21_N_2_O_5_	Yao (2018)
**92**	benzoic acid	C_7_H_6_O_2_	Wu (2016)
**93**	phenylmethanol	C_7_H_8_O	Wu (2016)
**94**	dibutyl phthalate	C_16_H_22_O_4_	Wu (2016)
**95**	furan-2-carboxylic acid	C_5_H_4_O_3_	Yao (2018)
**96**	β-sitosterol	C_29_H_50_O	Yao (2018)
**97**	β-daucosterol	C_35_H_60_O_6_	Yao (2018)

### Volatile Constituents

The species of the chemical constituents of essential oils has been studied for many years. They were isolated by steam distillation or supercritical fluid extraction, and analyzed by GC-MS. Volatile oils are generally composed of esters, hydrocarbons, terpenes and aromatic compounds. The 19 major compounds of essential oils are esters and terpenoids such as ethyl cinnamate, p-methoxycinnamate, pentadecane, δ-selinene, borneol, eucalyptol (Fan et al., 2005; Zhou et al., 2006; Zhang, 2007; Cui et al., 2008; Wang et al., 2009; Sutthanont et al., 2010; Liu et al., 2014; Luo et al., 2014; Raina and Abraham, 2016; Yang et al., 2018) **(** Table 4; Figure 2).

**TABLE 4:** Major compounds of essential oils of *K. galanga.*

No	Chemical constituent	Area						References
		Kerala and Karnataka (India)	Chiang Mai (Tailand)	Guangdong (China)	Guangxi (China)	Hainan (China)	Guizhou (China)	
1	borneol	1.0–2.4%	1.03%	0.17%	2.79%	—	—	Raina and Abraham, 2016; Sutthanont et al. (2010); Fan et al. (2005); Zhou et al. (2006); Zhang (2007)
				1.39%	1.62%			
2	β-pinene	0.1–0.3%	0.11%	0.12%	0.13%	—	0.01%	Raina and Abraham, 2016; Sutthanont et al. (2010); Zhou et al. (2006); Luo et al. (2014)
3	camphene	0.1–0.9	0.37	0.9	1.07	0.82	—	Raina and Abraham, 2016; Sutthanont et al. (2010); Zhou et al. (2006); Cui et al. (2008)
4	δ-carene	0.1–6.5	—	6.61	8.21	5.27	0.10	Raina and Abraham, 2016; Zhou et al. (2006); Cui et al. (2008); Luo et al. (2014)
5	eucalyptol	0.2–5.2	2.12	0.01	1.59	—	—	Raina and Abraham, 2016; Sutthanont et al. (2010); Fan et al. (2005); Zhou et al. (2006); Zhang (2007)
				0.56	0.16			
6	α-terpineol	0.1–0.3%	—	—	−0.23	—	—	Raina and Abraham, 2016; Zhang (2007)
7	p-cymene	0.1–1.1	—	0.65	0.79	—	0.01	Raina and Abraham, 2016; Zhou et al. (2006); Zhang (2007); Luo et al. (2014)
					0.93			
8	limonene	0.1–0.7	—	0.52	0.76	1.36	0.02	Raina and Abraham, 2016; Zhou et al. (2006); Zhang (2007); Cui et al. (2008); Luo et al. (2014)
					0.10			
9	p-methoxystyre-ne	—	—	0.46	0.78	—	—	Zhou et al. (2006)
10	τ-cadinene	0.3–0.5	—	0.15	1.25	—	0.15	Raina and Abraham, 2016; Fan et al. (2005); Zhang (2014)
11	δ-selinene	—	—	0.29	0.35	—	0.14	Zhou et al. (2006); Luo et al. (2014)
12	germacrene D	0.5–0.9	—	1.48	—	—	0.06	Raina and Abraham, 2016; Fan et al. (2005); Luo et al. (2014)
13	cyperene	—	—	0.63	1.464	—	0.95	Fan et al. (2005); Zhang (2007); Luo et al. (2014)
14	globulol	—	—	—	2.35	—	—	Zhang (2007)
15	ethyl cinnamate	11.5–26.6	—	5.27	27.74	19.32	—	Raina and Abraham, 2016; Fan et al. (2005); Zhou et al. (2006); Zhang (2007); Cui et al. (2008)
				23.68	28.30			
16	ethyl p-methoxycinn-amate	28.4–70.0	25.96	59.24	48.30	49.12	—	Raina and Abraham, 2016; Sutthanont et al. (2010); Fan et al. (2005); Zhou et al. (2006); Zhang (2007); Luo et al. (2014)
				59.96	33.84			
17	8-heptadecene	0.2–0.6	0.71	0.78	1.08	—	—	Raina and Abraham, 2016; Sutthanont et al. (2010); Fan et al. (2005); Zhang (2007)
18	pentadecane	6.0–16.5	26.1	21.67	14.85	15.02	—	Raina and Abraham, 2016; Sutthanont et al. (2010); Fan et al. (2005); Zhang (2007); Cui et al. (2008)
19	*Z*-9,11-dodecadien-1-ol acetate	—	—	0.99	—	—	—	Fan et al. (2005)

These essential oils show various promising pharmacological and therapeutic potentials, particularly, ethyl cinnamate and p-methoxycinnamate (Peter, 2004; Raina and Abraham, 2016). What’s more, *K. galanga.* has always been used as food flavoring and aromatic, due to its flavor and fragrances, which might be up to ethyl p-methoxycinnamate (Srivastava et al., 2019).

### Terpenoids

Terpenoids were the representative class of compounds isolated from *K. galanga*. To date, 26 terpenoids (**1–26**, Figure 3) have been isolated and identified, which included monoterpenoids, sesquiterpenoids and diterpenoids. Most of them were isopimarane type diterpenoids with the typical structural features of two double bonds of △^15(16)^, △^8(9)^ and/or △^8(14)^.

Among them, 3-caren-5-one (**1**) was a monoterpene ketone, which was isolated from methanolic extract of *K. galanga* firstly (Kiuchi et al., 1987). More recently, four new diterpenoids **6**, **19**, **20**, **26** were isolated and elucidated. Kaemgalangol A (**6**) was isolated from the chloroform fraction of methanol extract of *K. galanga*, and it was remarkable that **6** contained a rare 9,10-seco-isopimarane skeleton (Ningombam et al., 2018). From the hexane fraction of 95% ethanol extract of *K. galanga*, diterpenoids **19** and **20** were also identified (Tungcharoen et al., 2020). Compound **26** was isolated from the chloroform fraction of 75% ethanol extract of *K. galanga* (Yao, 2018).

### Phenolics

Phenolics (**27–41**, Figure 4) are compounds with a phenolic hydroxyl group (Li et al., 2017a; Hua et al., 2018). Depending on the existing literatures, 16 phenolic chemical constituents were found. Among them, **27–29** were hydroxybenzoic acids, and **35–37** were hydroxycinnamic derivatives. In addition, phenolic acids may be found in plants as in the form of glycosides (Masullo et al., 2015), such as **31** and **41** (Yao et al., 2018). Besides, **40** and **41** were first isolated and their structures were elucidated by the NMR, HR-MS and IR (Wu, 2016; Yao et al., 2018).

### Cyclic Dipeptides

Cyclic dipeptides (**42–57**, Figure 5) are formed by cyclization of two amino acids through peptide bonds. They are the simplest members in the most common cyclic peptide family found in nature. A total of 16 cyclic dipeptides have been reported (Yao, 2018).

### Flavonoids

The parent nucleus structure of flavonoids is 2-phenylchromone. The flavonoids isolated from *K. galanga* were all free monomers (**58–60**, Figure 5), and the substituents are usually methoxy and phenolic hydroxyl groups (Wu, 2016; Jiao et al., 2017). Kaempferol (**58**) (Chen et al., 2012) and luteolin (**59**) (Liu et al., 2018) had protective effects on lung injury by regulating multiple cellular pathways. Moreover, **58** (Schwarz et al., 2014) have been reported to exert anti-corona virus effects, indicating its potential in the treatment of COVID-19. Similarly, its relative, compound **59** could dose-dependently inhibit the SARS coronavirus cleavage activity with low micromole inhibitory activity (EC_50_ = 10.6 μM) (Yi et al., 2004), particularly, it could also inhibit the 3CLPro of SARS-CoV2 with IC_50_ value of 20.2 μM (Ryu et al., 2010). In silicon docking indicated that **59** could interact with a series of key targets of SARS-CoV-2 (3CLpro, PLpro, Spro and RdRp) to exert potential anti-corona virus activity (Yu et al., 2020).

### Diarylheptanoids

Diarylheptanoids (**61–68**, Figure 6) have a 1,7-diphenylheptane skeleton. Based on the skeleton, diarylheptanoids could be divided into linear and cyclic structural types. Linear diarylheptanoids occurred frequently in plants of Zingiberaceae family, and all the diarylheptanoids isolated from *K. galanga* were linear.

The first report of two novel sulfonated diarylheptanoid epimers focused on the identification of kaempsulfonic acid A (**67**) and B (**68**) (Wang et al., 2013). More recently, cyclic diarylheptanoids were isolated and elucidated. The two compounds, **62** and **61** were very similar to each other, while the difference was the substituents, **62** had one phenolic hydroxyl replaced by one glucosyl moiety. In addition, a linear diarylheptanoid **65** was isolated and elucidated (Yao, 2018; Yao et al., 2018).

### Fatty Acids and Esters

Fatty acid and esters were included in *K. galanga*, currently, 11 fatty acid and esters (**69–79**, Figure 6) have been analyzed and identified from *K. galanga* (Wu, 2016; Yao, 2018).

### Polysaccharides

Recently, the water-soluble polysaccharides (**80–88**, Figure 7) from *K. galanga*. (KGPs) were extracted and purified for the first time, and further investigated by different spectroscopic techniques such as HPGPC, FTIR, IC. Results showed that fucose (80), arabinose (81), xylose (82), rhamnose (83), mannose (84), galactose (85), glucose (86), glucuronic acid (87), and galacturonic acid (88) were the main components of KGPs, and their the molar ratio is 0.37: 3.12: 1.23: 3.09: 1.00: 6.39: 1.36: 0.91: 1.27, which significantly indicated that KGPs were heterogeneous acidic polysaccharides (Yang et al., 2018).

### Other Compounds

Apart from those chemical constituents mentioned above (**1–88**), *K. galanga* also contained other eight compounds (**89–97** Figure 7). Three pyroglutamic acids (**89–91**), two steroids (**96–97**) and three aromatic compounds (**92–95**) have been isolated and identified (Wu, 2016; Yao, 2018).

Molecular docking assay was used to investigate the effect of **92** as coronavirus polymerase (RdRp) inhibitor, and the results showed its potential anti-coronavirus activity with the binding energies showed −5.54 kcal/mol. Moreover, further studies are required to determine the potential uses of **92** in COVID-19 treatment (El-Aziz et al., 2020). Meanwhile, β-sitosterol (**96**) have been reported to have inhibitory activity against the SARS-CoV 3CLpro with IC_50_ value of 47.8 μg/ml (Lin et al., 2005).

### Elemental Composition

*K. galanga* was abounded with mineral elements K, P, Mg, Ca, Al, Fe, Na and Mn, and the content of K was the highest, amounting to 18,600 μg/g (Huang et al., 2012).

## Pharmacological Activities

*K. galanga* have gained much attention with its comprehensive pharmacological potential to treat a variety of human diseases. Modern pharmacological investigations have revealed that the extracts and natural products identified from *K. galanga* exhibited comprehensive bioactivities, including antitumor, antioxidant, anti-inflammatory and anti-tuberculosis, etc. Besides, the aqueous extract from its leaves have been reported to exert antinociceptive activity and anti-inflammatory activities in a dose dependently manner, supporting its traditional uses in the remedy of treat pain and mouth ulcer (Sulaiman et al., 2008). In addition, the kill of booklice by its essential oil, indicating its potential in the development of a natural insecticide and repellent for controlling stored grain pests (Liu et al., 2014). The more detailed pharmacological reviews were as follows.

### Antitumor Activity

According to the previous reports, the extracts and active components of *K. galanga* showed potential inhibitory effects on many types of tumors, such as gastric cancer, colon carcinogenesis, oral cancer and multiple myeloma. Although *K. galanga* preparations traditionally are used as an alternative medicine for tumor, there is little scientific evidence available about the use of *K. galanga* as an anticancer agent. Reports indicate that the anticancer signaling mechanisms of *K. galanga* extracts and compounds include inhibition of the growth of tumor cells, apoptosis and cytotoxicity, among others.

Multiple constituents isolated from *K. galanga* showed antitumor activity. It is reported that both *trans-*and *cis-*ethyl p-methoxycinnamate (**34**, **39**) could exert anti-carcinogeneic effect in an *in vitro* EBV assay with IC_50_ values of 5.5 and 9.5 μM (Xue and Chen, 2002). *Trans*-ethyl p-methoxycinnamate (**34**) was examined on HSC-3 and Ca922 lines by MTT assay. The MTT assay showed **34** could exert potent cytotoxicity in HSC-3 (IC_50_ = 0.075 mg/ml) and Ca922 (IC_50_ = 0.085 mg/ml) cell lines (Ichwan et al., 2019). In addition, **34** could also dose dependently induce apoptosis, and affected the cell cycle progress of the cell cycle of HepG2 cells (Liua et al., 2010). *Trans*-p-methoxycinnamic acid (**37**) (40 mg/kg b.w.) exhibited ameliorating anticancer effects in DMH-induced rat colon carcinogenesis by regulating of various processes, such as proliferation, invasion, angiogenesis, apoptosis and inflammation (Gunasekaran et al., 2019). The diarylheptanoid compounds sandaracopimaradiene-9*α*-ol (**10**), kaempulchraol I (**11**), kaempulchraol L (**14**) revealed anti-cancer effect in human HeLa (IC_50_ = 75.1, 74.2 and 76.5 μM, respectively) and HSC-2 cancer cells (IC_50_ = 69.9, 53.3 and 58.2 μM, respectively) by using MTT assay (Ningombam et al., 2018).

The essential oils from the *K. galanga* have displayed moderate antitumor activity. Flow cytometry (FCM) was used to evaluate the effect of volatile oil on cell cycle and apoptosis of MKN-45 cells. The growth inhibition rates of gastric cancer were 57.2, 28.0 and 5.0% respectively in the high-, medium-, and low-dose volatile oil-treated groups (1.56, 0.78, 0.39 g/d), and the gastric cancer cells (MKN-45 cells) were arrested at G0/G1phase. The results showed the high-dose volatile oil-treated group was effective for inhibiting the growth of gastric cancer by comparing to cyclophospha (CTX)-treated group (78.9%) (Xiao et al., 2006). The ethanolic extract of *K. galanga* and its major bioactive constituent *trans*-ethyl p-methoxycinnamate (**34**) could exert cytotoxic activity against cholangiocarcinoma cells (CL-6). The ethanolic extract inhibited CL-6 cell growth at doses of 125 and 250 μg/ml, with 80 and 94% inhibitory, and IC_50_ values of 64.2 and 49.19 μg/ml, respectively (Amuamuta et al., 2017). Recently, the methanolic and acetonic extracts of *K. galanga* leaves have been reported to exert moderate cytotoxic activities (LC_50_ = 4.78 and 0.11 μg/ml, respectively) in the brine shrimp lethality bioassay (Rahman et al., 2019). The water-soluble polysaccharides isolated from *K. galanga* could inhibit the growth of H22 solid tumors, while exert protective effects on the thymus and spleen of solid tumor bearing mice (Yang et al., 2018).

### Anti-Inflammatory Activity

The traditional applications of *K. galanga* in the remedy of abdominal pains and toothache are mostly depend on its anti-inflammatory effects. The mechanism behind the anti-inflammatory action of *K. galanga* is associated with the presence of bioactive metabolites by inhibiting the release of inflammatory factors.

The anti-inflammatory effect of *trans*-ethyl p-methoxycinnamate (**34**) was assessed using the cotton pellet granuloma assay in rats *in vivo*, and *in vitro* using the human macrophage cell line (U937). It strongly inhibited granuloma tissue formation in rats and the release of IL-1 and TNF-*α*, which were significantly inhibited in both *in vivo* and *in vitro* models (Umar et al., 2014). Kaempferol (**58**) exerted potent inhibitory activity on HMC-1 mast cell-mediated inflammatory response stimulated by lipopolysaccharide (LPS) demonstrated by MTT assay. The release of IL-6, IL-8, IL-1*β* and TNF-*α* significantly decreased at the dose of 40 μmol/L (Zhou et al., 2015). Moreover, diarylheptanoids **61**, **63**, **65**, **66**, have been reported to inhibit nitric oxide (NO) production on LPS-induced macrophage RAW264.7 cell lines with IC_50_ values of 27.85, 46.98, 26.98 and 17.26 μM, respectively (Yao et al., 2018).

The leaves of *K. galanga* have been reported to exert potent anti-inflammatory activity in a modified carrageenan-induced paw-edema test, supporting its traditional applications of ulcers and pains (Sulaiman et al., 2008). The various extracts of *K. galanga* exerted anti-inflammatory effects *in vivo*. In carrageenan induced acute inflammation test, the successive petroleum ether fraction (SPEF) showed 39.16% effect (300 mg/kg b.w., p.o.), followed by the successive ethyl acetate fraction (SEAF), alcohol fraction (SAF) and alcoholic extract with respective 10.0, 22.5 and 5.0% effects. In adjuvant-induced chronic inflammation test, the SPEF and diclofenac extract obviously reduced inflammation (5 and 100 mg/kg b.w., p.o., 7 days) (Jagadish et al., 2016).

### Anti-Oxidant Activity

The anti-oxidant activity is an important value for the further development of natural products, since oxidation reactions are associated with many diseases (Liu and Ng, 2000). In the past few years, crude extracts with anti-oxidant activity from *K. galanga* has been evaluated using several methods as follows.

It is reported that the essential oil extracted by ultrasound-enhanced subcritical water extraction (USWE) exerted significant DPPH, free radical and superoxide anion radical scavenging effects, suggesting its strong anti-oxidant effects (Ma et al., 2015). The methanolic extract of *K. galanga* showed high antioxidant activity in DPPH, ABTS, and NO scavenging assays (IC_50_ = 16.58, 8.24 and 38.16 μg/ml, respectively) (Ali et al., 2018). Further, the *K. galanga* leaves showed weakly antioxidant activity in DPPH scavenging assay, and IC_50_ values were 611.82 and 702.79 μg/ml, respectively (Rahman et al., 2019). The antioxidant activity of various extracts of *K. galanga* were tested by DPPH and ABTS assays respectively. The results showed that *K. galanga* had good antioxidant activity, among the five extracts, the activity of chloroform fraction was the best, and its SC_50_ on DPPH and ABTS were about 4 and 2 times that of the positive control (V_C_) respectively, followed by the ethyl acetate, n-butanol fraction, while petroleum ether fraction was poor and the water fraction was basically inactive (Xiang et al., 2018).

### Insecticidal and Repellent Activity

The methanolic extract and essential oil of *K. galanga* rhizome, as well as their isolates *trans*-ethyl p-methoxycinnamate (**34**) and *trans*-ethyl cinnamate (**38**) exhibited strong insecticidal and repellent properties.

Two compounds **34** and **38** with excellent nematicide activity had been obtained from petroleum hexane extracts. After treatment 72 h, the LC_50_ of **34** against *Meloidotyne incongnita*, *Bursaphelenchus xylophilus*, *Ditylenchus destructor*, *M. eloidogyne hainensis*, *M. enterolobii* were 1.49, 2.81, 10.09, 26.67 and 14.47 mg/L. The LC_50_ values of **38** against *M. incongnita*, *B. xylophilus*, *D. destructor*, *M. eloidogyne hainensis*, *M. enterolobii* were 17.79, 29.70, 43.21, 57.64 and 36.94 mg/L, respectively (Zhang et al., 2010). **34** and **38** also had potent insecticidal activity against the larvae of polyphagous insect *Spodoptera littoralis* (Noctuidae) (LD_50_ = 0.47 and 0.65 μg/mL, respectively) (Pandji et al., 1993).

The essential oil, and its main constituents, **38**, **34** and **39** showed contact toxicity against the booklouse *Liposcelis bostrychophila* Badonnel. Among them, **38** was the most effective with LC_50_ value of 21.4 g/cm^2^, **34**, **39** and the essential oil exhibited moderately effects with LC_50_ value of 44.6 and 43.4 68.6 g/cm^2^, respectively. In addition, fumigant toxicity (LC_50_ = 1.5 mg/L air) of the essential oil against the booklouse also was observed (Liu et al., 2014). The essential oil as well as **34** and **38** showed nematicidal activity against the cereal cyst nematode with LC_50_ value of 91.78, 83.04 and 100.60 μg/ml, respectively, while borneol and 1, 8-cineole only showed slight nematicidal toxicity (LC_50_ = 734.89 and 921.21 μg/ml, respectively) (Li et al., 2017b).

The toxicity of the methanol extracts against *Bursaphelenchus xylophilus* and *Meloidotyne incongnita* were tested. The results showed that the mortality of extracts from *K. galanga* against *B. xylophilus* and *M. incongnita* with 100% mortality at 1,000 mg/L after 24 h (Choi et al., 2006).

### Antimicrobial Activity

*Trans*-ethyl p-methoxycinnamate (**34**) exerted potent antibacterial activity against *Pseudomonas aeruginosa*, *Escherichia coli*, *Staphyloccocus aureus*, *Aspergillus niger* and *Monilia albican* in disk diffusion and test tube experiments, and the MIC values were 0.625 1.25, 2.5, 2.5 and 10 mg/ML, respectively (Han et al., 2011). The essential oil of *K. galanga* showed potent antimicrobial activity against *Candida albicans* (fungus); *Staphylococcus aureus* ATCC 25923, *S. faecalis* and *Bacillus subtilis* (three Gram-positive bacteria); *Salmonella typhi*, *Shigella flexneri*, *Escherichia coli* ATCC 25922 (three Gram-negative bacteria) in agar disc diffusion test, with the inhibition zones was 12–16 mm and 8–12 mm against Gram-positive and Gram-negative bacteria respectively, while it potently inhibited *C*. *albicans* with an inhibition zone of 31 mm, comparing to that (25 mm) of standard antifungal (Clotrimazole) (Tewtrakul et al., 2005). Similarly, agar well diffusion test was employed to assess antifungal potential of ethanolic extract of *K. galanga*, and the results showed a potent antifungal effect of this extract against *Malassezia* spp. (MIC = 5 mg/ml) (Parjo et al., 2018).

### Antidiabetic Activity

Diabetes has become the third major non-infective disease threatening human health after cardiovascular disease and tumor (World Health Organization, 2013). The effect of kaempferol (**58**) on the correlation factors of chronic complications of type 2 diabetic rats was observed. Rats in administration group were given respective drug (50, 100, 200 mg/kg) every day, and set the model, normal control, and positive control (metformin hydrochloride 0.2 g/kg) groups. After 10 weeks, compared with diabetic model group, **58** administration could reduce blood lipid levels, along with reducing MDA, AR, TNF-α, and IL-6 levels and increasing SOD levels. Moreover, **58** could prevent and treat the chronic complications of type 2 diabetic rats by reducing blood glucose, insulin resistance, reducing the AR pathway as well as anti-oxidation and anti-inflammation. The antidiabetic activity of **58** was comparable to that of positive control at the dose of 200 mg/kg (Wu et al., 2015).

### Anti-Tuberculosis Activity

The anti-tuberculosis effect of **34** was determined by resazurin microtitre assay (REMA) on *Mycobacterium tuberculosis* H37Ra and H37Rv strains. The results demonstrated that **34** had a significant anti-tuberculosis activity, and its MIC values were in the range of 0.242–0.485 mM. This study showed *K. galanga* and its isolate **34** had anti-tuberculosis effects, however, the molecular mechanisms of action of **34** should be further explored by in-depth studies and clinical trials (Lakshmanan et al., 2011).

### Vasodilatory Activity

Previous reports have shown that *trans*-ethyl cinnamate (**38**) could exert vasorelaxant activity, which was in line with traditional role of *K. galanga* in the treatment of high blood pressure. It could dose dependently inhibit the tonic contractions induced by high concentrations of K^+^ and phenylephrine (PE) (IC_50_ = 0.3 ± 0.05 and 0.38 ± 0.04 mM, respectively). Mechanistic studies revealed that its vasorelaxant activity could be attributed to the inhibition of influx of Ca^2+^ into vascular cells and the release of prostacyclin and NO from the endothelial cells. Hence, the traditional use of the herb in treating hypertention may be explained well by the vasorelaxant activity of **38** (Othman et al., 2002). In the anaesthetized rats, the dichloromethane extract of *K. galanga* could exert vasorelaxant activity by lowering the basal mean arterial pressure (MAP). Moreover, the active compound, **38** was identified by bioassay-guided fractionation and isolation (Othman et al., 2006).

### Sedative Activity

The hexane extract of *K. galanga* demonstrated potent sedative effects (1.5 and 10 mg) by reducing the activity of locomotor. Moreover, *trans*-ethyl p-methoxycinnamate (**34**) and *trans*-ethyl cinnamate (**38**) as well as showed significant sedative activity (14 and 12 μg) (Huang et al., 2008). The acetone extract of *K. galanga* exerted sedative activity at the dose of 200 mg/kg in mice (b.w., p.o.) (Ali et al., 2015).

### Anti-Angiogenic Activity

The anti-angiogenic effects of ethanol extract, *trans*-ethyl p-methoxycinnamate (**34**) and kaempferol (**58**) of *K. galanga* exhibited potent anti-angiogenic effect assessed by zebrafish angiogenic assay. Further investigations for action mechanism of **34** indicated that it inhibited the migration and tube formation of human umbilical vein endothelial cells *in vitro*, and blocked vessel formation induced by bFGF on Matrigel plug assay *in vivo* (He et al., 2012).

### Anti-Osteoporosis Activity

Kaempferol (**58**) showed inhibitory effects of osteoclastogenesis in the autophagy inhibition process of RAW 264.7 cells in the presence of 50 μM, and obviously inhibited the expression of p62/SQSTM1. Moreover, the potential role of **58** for the treatment of bone metabolism disorders could be explored through in-depth study of the role of p62/SQSTM1 in autophagy (Chang-Ju et al., 2018). Kaempferide (**60**) could prevent osteolysis induced by titanium particle and inhibit osteoclast genesis in mice at 12.5 μM, indicating a potential agent with anti-osteoporosis activity (Jiao et al., 2017).

### Antithrombotic Effect

The ethanolic extract of *K. galanga* was orally administered (7, 14 and 28 mg/20 g b.w.) in a mouse thrombotic model induced by collagen-epinephrine. Bleeding time prolongation and the survival rate of mice was observed after 7 days extract pre-treatment. The results showed the greatest antithrombotic potency of *K. galanga* extract had similarities with the positive control (aspirin) at its highest dose (28 mg/20 g b.w.). Thus, the herb had great chance to be an antithrombotic agent in further studies (Saputri and Avatara, 2018).

### Hypopigmentary Effect

Kaempferol (**58**) was investigated for the effect on tyrosinase activity, melanin content, and cell proliferation in human normal melanocytes. The effects of various concentration (1–100 μM) of kaempferol upon proliferation, melanin synthesis and tyrosinase activity in human normal melanocytes were observed. The results showed **58** could strongly inhibit tyrosinase activity and melanin content of melanocyte without more toxicity or adverse side effect on proliferation of melanocytes, and suggested **58** was a promising tyrosinase inhibitor (Shang et al., 2011).

### Anti-Sunburn Activity

It was reported that *trans*-ethyl p-methoxycinnamate (**34**) could protect skin from sunburn. In order to investigated anti-sunburn activity of **34**, *in vitro* percutaneous solution was established, and the percutaneous absorption of **34** was studied. Modified Franz diffusion cells were used for *in vitro* permeation studies, and the nude mouse skin was used as transdermal barrier. The concentration of **34** in the receptor solution was determined by HPLC, and it also displayed a certain extent of sunscreen efficacy. The results showed that, accumulative permeation amount of **34** within 10 h was 0.2949 mg/cm^2^ and indicated it was suitable for the development of natural sunscreen cosmetic products (Li et al., 2013).

The ethanolic rhizome extract of *K. galanga* and its main constituent **34** were evaluated for their UV protective properties. The results demonstrated *K. galanga* presented high UVB protection with SPF range of 8.57–22.34 μg/ml, and its main constituent **34** also demonstrated UV protective effect (Panyakaew et al., 2020).

## Conclusion and Perspective

This review summarizes the latest researches of different extracts and active compounds of *K. galanga* in the fields of ethnomedicine uses, phytochemistry, toxicology and pharmacology. As stated above, the ninety-seven bioactive phytochemicals including terpenoids, phenolics, cyclic dipeptides, flavonoids, diarylheptanoids, fatty acids and esters, and others, have been isolated and identified from *K. galanga*, suggesting the presence of potential structural diversity of *K. galanga*, among them, isopimarane-type diterpenoids as the mainly characteristic constituents. Furthermore, numerous pharmacological studies have revealed that various crude extracts and some chemical components exerted multiple biological activities, in particular, antitumor, anti-inflammatory and anti-oxidant activities.

Although phytochemistry studies have isolated some compounds from the rhizomes of this plant, no study has documented the constituents separated from the leaves. Thus, the chemical studies on the leaves of this plant are necessary to strengthen. Besides, new compounds are need to be explored for enriching material basis of *K. galanga.*

Most pharmacological studies of *K. galanga* concentrated on the activities of its crude extracts, particularly volatile oil and ethanol extract. However, the underlying mechanisms of activities and exact chemical constituents are still little knowledge. Therefore, further elucidating the relationships of pharmacological mechanisms of bioactive constituents are still required. Gratefully, an emerging technology, DNA-encoded library (DEL) and especially the natural product DNA-encoded library (*n*DEL) has already showed their potential in identification of protein targets of natural products, thus could be used to handle this issue (Ma et al., 2019; Xie et al., 2020). Compounds that isolated from *K. galanga* could be efficiently annotated with unique DNA tags by using the *n*DEL technology to form a *K. galanga* focused *n*DEL. Screening of the *K. galanga* focused *n*DEL against various protein targets will definitely help to illuminate the target network of *K. galanga* in the future.

With regard to the safety profile of *K. galanga*, existing studies have provided only limited information. More systematic toxicology studies are still needed to be carried out in the future on the extracts and purified compounds of *K. galanga*.

For further improving the species, the following aspects also need to be paid attention to. Innovative breeding designs supported by information on the genomic resources and appropriate technologies could play a potential role to realize stable growth in *K. galanga* productivity and quality (Bohra et al., 2020). In addition, to develop an agrotechnology to commercialize the production of *K. galanga* and bioprospect in *K. galanga* is required to identify secondary metabolite and develop novel technologies to overcome some diseases (Jnanesha and Kumar, 2018). On the other hands, clarifying biosynthetic pathways of bioactive natural products will make a significant contribution to pave the way for their manufacture. (Thomford et al., 2018; Gao and Lei, 2020). Moreover, plant proteomics of *K. galanga* could open new perspectives for ethnobotanical and phytomedicine research purposes, indicating the use of medicinal plants for the treatment of certain diseases (Pedrete, et al., 2019).

In terms of quality control, the information about the cultivation environment, cultivars, processing, transportation, storage time, and quantitative studies of the index components are scarcely in the existing studies. It is worth noting that the dual quality control, chemical benchmark and effect benchmark has been generally accepted (Li et al., 2019; Zhu et al., 2019). Therefore, the quality standard of it could be supported by the effect benchmark. Moreover, the effect benchmark offer basis to Q-biomarker research strategy, which could provide reference of methodology for the quality control study of *K. galanga* (Geng et al., 2019; Lu et al., 2020).

In summary, this review provides a comprehensive analysis on ethnomedicinal uses, phytochemistry, pharmacology and toxicology of *K. galanga*, and proposed future research directions. Based on this, we hope to highlight the potential value of *K. galanga* and provide some new research directions in further studies.
